# Plasminogen activator inhibitor (PAI)-1 suppresses inhibition of gastric emptying by cholecystokinin (CCK) in mice^☆^

**DOI:** 10.1016/j.regpep.2013.06.005

**Published:** 2013-08-10

**Authors:** Joanne Gamble, Susan Kenny, Graham J. Dockray

**Affiliations:** Physiological Laboratory, Institute of Translational Medicine, University of Liverpool, Liverpool, UK

**Keywords:** CCK, cholecystokinin, G17, gastrin, PAI, plasminogen activator inhibitor, PAI-1, CCK, Gastric emptying

## Abstract

The intestinal hormone cholecystokinin (CCK) delays gastric emptying and inhibits food intake by actions on vagal afferent neurons. Recent studies suggest plasminogen activator inhibitor (PAI)-1 suppresses the effect of CCK on food intake. In this study we asked whether PAI-1 also modulated CCK effects on gastric emptying. Five minute gastric emptying of liquid test meals was studied in conscious wild type mice (C57BL/6) and in transgenic mice over-expressing PAI-1 in gastric parietal cells (PAI-1H/Kβ mice), or null for PAI-1. The effects of exogenous PAI-1 and CCK8s on gastric emptying were studied after *ip* administration. Intragastric peptone delayed gastric emptying in C57BL/6 mice by a mechanism sensitive to the CCK-1 receptor antagonist lorglumide. Peptone did not delay gastric emptying in PAI-1-H/Kβ mice. Exogenous CCK delayed gastric emptying of a control test meal in C57BL/6 mice and this was attenuated by administration of PAI-1; exogenous CCK had no effect on emptying in PAI-1-H/Kβ mice. Prior administration of gastrin to increase gastric PAI-1 inhibited CCK-dependent effects on gastric emptying in C57BL/6 mice but not in PAI-1 null mice. Thus, both endogenous and exogenous PAI-1 inhibit the effects of CCK (whether exogenous or endogenous) on gastric emptying. The data are compatible with emerging evidence that gastric PAI-1 modulates vagal effects of CCK.

## Highlights

• Cholecystokinin (CCK) inhibits gastric emptying and food intake.
• PAI-1 inhibits effects of CCK on food intake.
• We hypothesised that PAI-1 also modulates gastric emptying.
• Both endogenous and exogenous PAI-1 attenuated the effect of CCK on gastric emptying.
• Gastric PAI-1 is therefore a modulator of CCK inhibition of gastric emptying.

## 1 Introduction

The delivery of liquid gastric contents to the duodenum is determined by their chemical composition. In particular, gastric emptying is delayed by protein and fat content, osmolarity, viscosity and acid. A number of different neuro-endocrine control mechanisms have been identified that act either to change the pressure difference across the pylorus, for example by relaxation of the gastric corpus, or by changing the resistance to flow across the pylorus. The role of the intestinal hormone cholecystokinin (CCK) as a physiological regulator of the gastric emptying of protein and fat has been relatively well studied since its discovery in the mid-1970s [1–5].

Dietary protein and fatty acids with a chain length greater than C12 release CCK which in turn regulates protein and lipid digestion by stimulating delivery to the duodenum of bile salts (through stimulation of gall bladder contraction) and pancreatic enzymes (through stimulation of pancreatic acinar cell exocytosis) [6]. At the same time CCK inhibits gastric emptying and food intake thereby matching delivery of food to the duodenum with the capacity for its digestion. There is now considerable evidence that the latter actions of CCK are mediated *via* stimulation of vagal afferent neurons which express the CCK-1 receptor [7]. In particular, inhibition of gastric emptying occurs through activation of vago-vagal reflexes leading to relaxation of the gastric corpus, and perhaps also increased resistance to flow across the pylorus [2,3]. Recent work suggests a number of different mechanisms that modulate vagal responses to CCK, including potentiation by leptin and inhibition by ghrelin [8–10].

Plasminogen activator inhibitor (PAI)-1 is normally expressed in gastric parietal cells and ECL cells and there is evidence of increased expression in response to high plasma gastrin concentrations [11] and *Helicobacter pylori* infection [12,13]. In order to study the function of PAI-1 in the stomach, we recently reported the generation of transgenic mice over-expressing PAI-1 in gastric parietal cells using a promoter sequence of approximately 1 kb of the H/K-ATPase β-subunit. Interestingly, these mice were moderately obese, hyperphagic and insensitive to the satiety action of CCK; moreover experiments in rat nodose ganglia *in vitro* indicated that the excitatory effect of CCK on vagal afferent neurons was inhibited by PAI-1 [14]. On the basis of these findings, we have now hypothesised that PAI-1 also inhibits the effect of CCK on gastric emptying. We report here that PAI-1-H/Kβ mice exhibit resistance to the effects of both endogenous and exogenous CCK in delaying gastric emptying in mice.

## 2 Materials and methods

### 2.1 Mice

Male mice, 10–13 weeks of age, were used for all studies. They were maintained on a 12:12 h light/dark cycle. Transgenic mice on a C57BL/6 background exhibiting targeted expression of PAI-1 in gastric partial cells using 1.1 kb of the proximal promote of H^ +^/K^ +^ ATPase β-subunit coupled to the coding sequence of mouse PAI-1 (i.e. PAI-1-H/Kβ mice) have previously been described [14]. Wild type C57BL/6 mice were obtained from Charles River (MA, USA) and mice null for PAI-1 were obtained from Jackson Laboratories (Maine, USA). All experiments were approved by the University of Liverpool Animal Welfare Committee and were conducted in compliance with the UK Animals (Scientific Procedures) Act, 1986.

### 2.2 Materials

Methyl cellulose, meat peptone (Primatone) and phenol red were obtained from Sigma-Aldridge (Gillingham, Dorset, UK); camostat mesilate (FOY305), sulphated CCK octapeptide (CCK8s) and unsulphated heptadecapeptide gastrin (G17ns) were obtained from Tocris Biosciences (Bristol, UK). The CCK-1 receptor antagonist lorglumide was a kind gift from Dr Massimo D'Amato (Rotta Research Laboratories, Milan, Italy). Stabilised human PAI-1 was obtained from Calbiochem (Hertfordshire, UK) [15].

### 2.3 Gastric emptying

Mice were fasted overnight and water was removed 1 h prior to procedures. Animals received intraperitoneal injections (100 μl; CCK8s or PAI-1 at 2.5 nmol/kg in saline, or saline alone) 5 min prior to gastric emptying studies. In some experiments mice received gastrin (20 nmol/kg) 6 h prior to gastric emptying studies. Mice received liquid test meals of either methyl cellulose (1.5% w/v in distilled water containing 50 mg/l phenol red) or peptone (4.5% w/v in methyl cellulose solution) at a volume of 600 μl by gavage. In studies of FOY305, animals were pretreated by gavage (100 μl, 100 mg/kg) 5 min before the test meal. All test meals were pre-warmed to 37 °C. Mice were culled 5 min after gavage by rising CO_2_ followed by cervical dislocation. An abdominal incision was made, the pylorus and oesophageal junctions were ligated, the stomach securely removed and the gastric contents collected into Eppendorf tubes and centrifuged (12,000 rpm, 5 min). The supernatant was collected, the volume measured and samples alkalinised with NaOH and absorbance determined at 550 nm. Samples of methyl cellulose and peptone test meals were used as controls. Gastric emptying was calculated as described by Debas et al. [1].

### 2.4 PAI-1 ELISA

Blood was obtained from fasted C57BL/6 mice, or PAI-1 null mice, between 15.00 and 16.00 h *via* cardiac puncture, collected in 0.1 M tri-sodium citrate, centrifuged and concentrations of plasma PAI-1 were determined by ELISA (Molecular Innovations, MI, USA) according to manufacturer's instructions. The assay was validated by demonstrating (a) undetectable PAI-1 in plasma from PAI-1 null mice, and (b) parallel dilution curves for standard PAI-1 and samples from C57BL/6 mice with high endogenous PAI-1.

### 2.5 Statistics

Data are expressed as means ± S.E.; comparisons made by *t* test for experiments involving a single experimental variable, or ANOVA for multiple comparisons using Bonferroni correction, and were considered significant at p < 0.05.

## 3 Results

### 3.1 Peptone delays gastric emptying in C57BL/6 but not PAI-1-H/Kβ mice

**Fig. 1:**
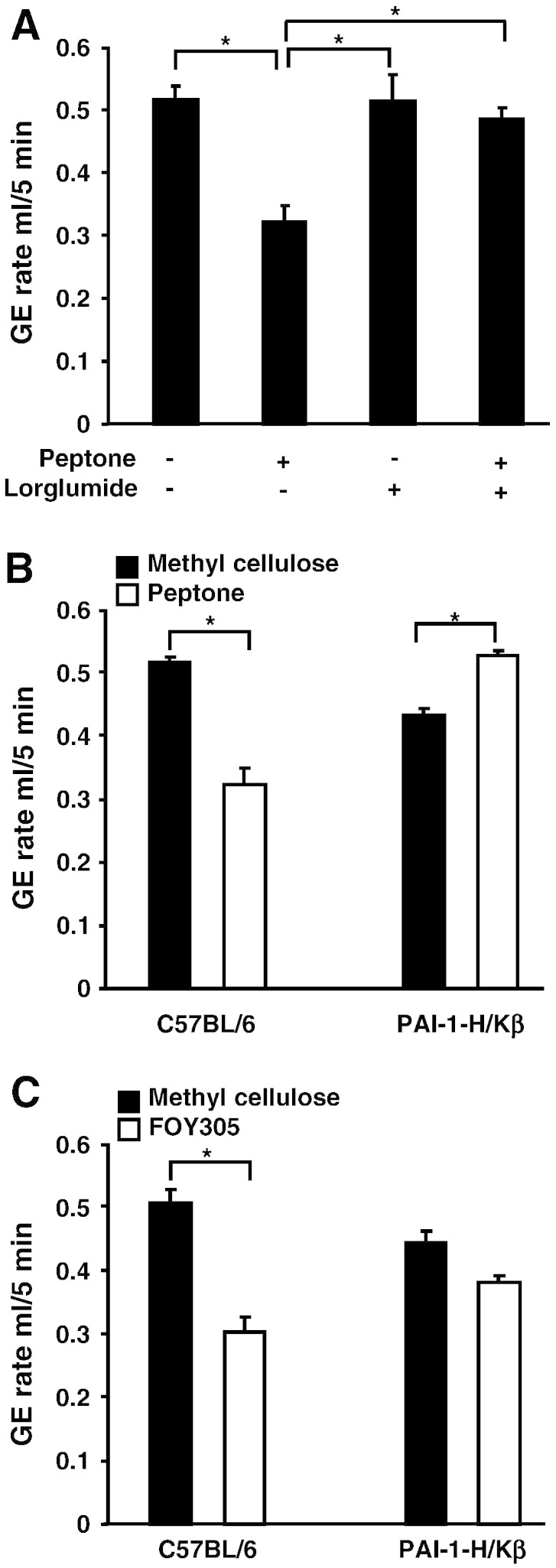
Peptone delays gastric emptying in C57BL/6 mice but not in PAI-1-H/Kβ mice. A, Peptone (5% w/v in 1.5% w/v methyl cellulose) inhibits gastric emptying compared with methyl cellulose alone in C57BL/6 mice and the CCK-1 receptor antagonist, lorglumide (4 mg/kg, *ip*) reverses this (n = 3–6; *, p < 0.05, ANOVA). B, Inhibition of gastric emptying by peptone in C57BL/6 mice (n = 17; *, p < 0.05, *t* test) but not in PAI-1-H/Kβ mice (n = 5). C, Pretreatment by gavage 5 min prior to test meals with the CCK-releasing agent, camostat mesilate (FOY305, 100 mg/kg) significantly delays gastric emptying of methyl cellulose in C57BL/6 but not in PAI-1-H/Kβ mice (n = 4–7; *p < 0.05, *t* test).

In initial experiments, we sought to adapt methods to the mouse that had previously used to study the role of endogenous CCK on gastric emptying in rats [3,16]. Thus over 85% of a control test meal of methyl cellulose emptied in 5 min following intragastric administration. In contrast, peptone dissolved in the methyl cellulose solution significantly delayed gastric emptying compared with methyl cellulose alone in C57BL/6 mice (Fig. 1A). The action of peptone was reversed by prior administration of the CCK-1 receptor antagonist lorglumide, consistent with a role for endogenous CCK in mediating the action of peptone on gastric emptying in wild type mice (Fig. 1A). However, in PAI-1-H/Kβ mice, peptone did not inhibit gastric emptying compared with methyl cellulose, and in fact emptied slightly more rapidly (Fig. 1B). Moreover, a different type of CCK-releasing meal, namely prior administration of FOY305 [17], also significantly delayed gastric emptying of methyl cellulose in C57BL/6 mice but not PAI-1H/Kβ mice compatible with the conclusion that over-expression of PAI-1 in gastric parietal cells inhibits delayed gastric emptying by endogenous CCK (Fig. 1C).

### 3.2 PAI-1 attenuates the action of exogenous CCK on gastric emptying

**Fig. 2:**
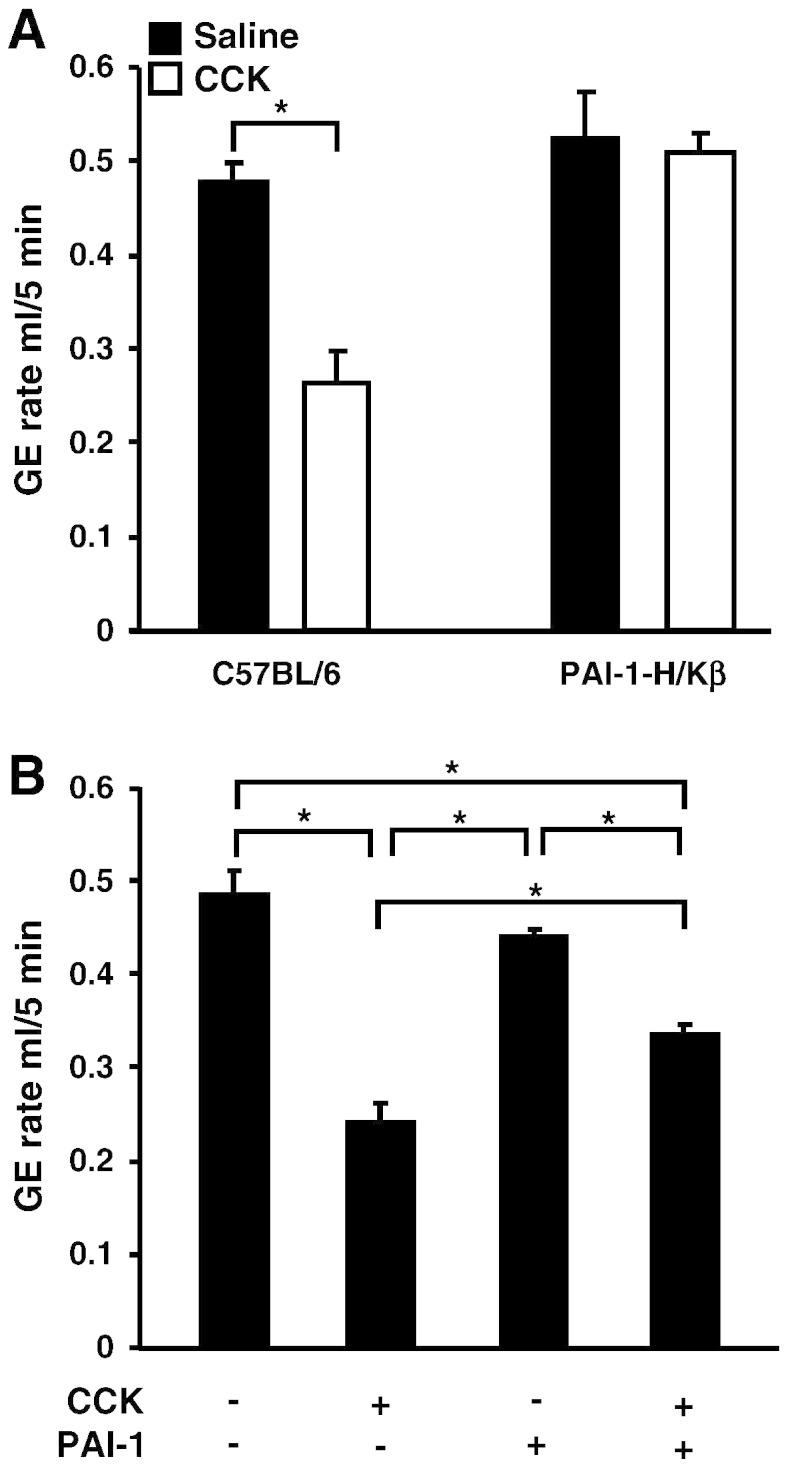
Exogenous CCK delays gastric emptying in C57BL/6 but not in PAI-1-H/Kβ mice. A, In C57BL/6, but not in PAI-1-H/Kβ, mice CCK8s (2.5 nmol/kg, 100 μl, *ip*) inhibits gastric emptying of methyl cellulose (n = 4–5; *, p < 0.05, *t* test). B, The action of exogenous CCK8s on gastric emptying in C57BL/6 mice is partially inhibited by exogenous PAI-1 (2.5 nmol/kg, *ip*) (n = 5; *, p < 0.05, ANOVA).

To determine whether PAI-1-H/Kβ mice were also resistant to the effects of exogenous CCK, we then studied the action of *ip* CCK8s (2.5 nmol/kg) on gastric emptying of a control test meal. At the dose used, there was approximately 50% inhibition of emptying of methyl cellulose in C57BL/6 mice. However, in PAI-1-H/Kβ mice there was no significant difference in the emptying of the control test meal after CCK8s compared with *ip* saline (Fig. 2A). We then asked whether exogenous PAI-1 also inhibited the effect of CCK on gastric emptying. In C57BL/6 mice, the inhibition of emptying of methyl cellulose in response to CCK8s was partially reversed by prior administration of PAI-1 (2.5 nmol/kg) (Fig. 2B). Moreover, *ip* administration of PAI-1 to C57BL/6 mice also accelerated the emptying of a peptone test meal compatible with inhibition of endogenous as well as exogenous CCK on gastric emptying (*ip* saline: 0.32 ± 0.02 ml of peptone emptied; *ip* PAI-1: 0.55 ± 0.02 ml emptied; *t* test, p < 0.05).

### 3.3 Increases in wild type PAI-1 reverse CCK-delayed gastric emptying

**Fig. 3:**
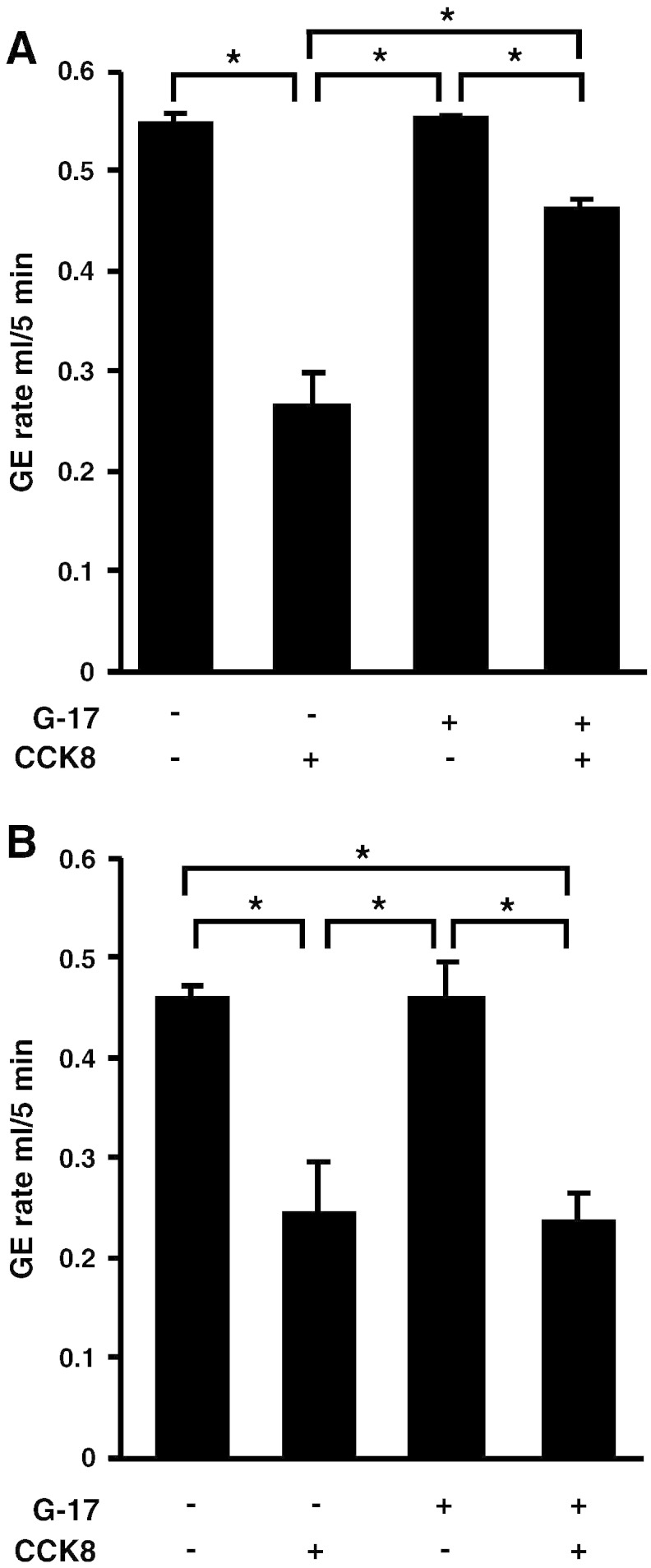
Pretreatment with gastrin (G17ns) reverses the effect of CCK on gastric emptying in C57BL/6 mice but not in PAI-1^−/−^ mice. A, Pretreatment with G17ns (20 nmol/kg, *ip*, 6 h previously), which significantly increases plasma PAI-1, inhibits the effect of CCK8s (2.5 nmol/kg, *ip*) on gastric emptying of methyl cellulose in C57BL/6 mice (n = 6; *, p < 0.05, ANOVA). B, Pretreatment with G17ns has no effect on the inhibition of gastric emptying of methyl cellulose in response to CCK8s in PAI-1^−/−^ mice (n = 6; *, p < 0.05 ANOVA).

It is known that PAI-1 expression is highly regulated, and elevated plasma gastrin is associated with increased expression in gastric epithelial cells notably parietal cells and ECL cells [11]. We therefore asked whether prior administration of gastrin to increase gastric PAI-1 might modulate CCK-effects on gastric emptying. There was increased plasma PAI-1 in C57BL/6 mice 6 h following *ip* administration of G17ns (20 nmol/kg) compared with saline (*ip* saline: 4.6 ± 0.6 ng/ml; G17ns: 9.8 ± 1.0 ng/ml; *t* test, p < 0.05); there was no significant difference in plasma PAI-1 after acute administration of CCK. In C57BL/6 mice, prior administration of G17ns significantly reversed the effect of subsequent administration of CCK8s in delaying emptying of methyl cellulose (Fig. 3A). Strikingly, however, in PAI-1 null mice, CCK inhibition of gastric emptying was maintained after prior treatment with G17 indicating a role for endogenous PAI-1 in mediating the effect of gastrin (Fig. 3B).

## 4 Discussion

The main finding of this study is that PAI-1 suppresses the inhibition of gastric emptying by CCK in mice. It is well recognised that CCK stimulates vagal afferent neurons resulting in delayed nutrient delivery to the small intestine by inhibition of both gastric emptying and food intake [7]. Previous studies have shown that PAI-1 suppresses CCK-inhibition of food intake, and have provided evidence that it also inhibits CCK-stimulation of vagal afferent neurons [14]. The present study was therefore undertaken to examine the hypothesis that PAI-1 also suppressed CCK inhibition of gastric emptying. The data derived from studies in transgenic mice over-expressing PAI-1 in the stomach, in PAI-1 null mice, and in wild type mice receiving exogenous CCK or PAI-1, all indicate that PAI-1 (whether exogenous or endogenous) inhibits the effect of CCK (both endogenous and exogenous) on gastric emptying. Thus PAI-1 should now be considered a putative modulator of CCK effects on gastric emptying in health and disease.

There is widespread expression of PAI-1 in many different cells including platelets, endothelial cells, hepatocytes, macrophages, monocytes, adipocytes and adipose stromal cells. Expression is increased in response to inflammation, sepsis, TGFβ and plasma concentrations are elevated in obesity [18–20]. In the stomach, there is expression of PAI-1 in both epithelial and stromal cells, and increased expression in parietal and ECL cells has been reported in response to gastrin [11] and *H. pylori* [12,13]. In PAI-1-H/Kβ mice targeted expression of PAI-1 to parietal cells was achieved using a promoter sequence of approximately 1 kb of the H/K-ATPase β-subunit which has been well characterised for this purpose. In qPCR, primers specific for the transgene sequence confirm selective expression in the stomach, while qPCR primers measuring total tissue PAI-1 mRNA abundance (i.e. transgene plus wild-type product) indicated an approximately 3-fold increase in gastric mRNA abundance. These animals exhibit approximately 25% higher food intake and moderate life-long obesity; moreover the hyperphagia in these animals is at least partly a consequence of insensitivity to CCK [14]. The increase in gastric PAI-1 mRNA abundance in PAI-1-H/Kβ mice is comparable to the changes seen in hypergastrinaemia, so that the insensitivity exhibited by these mice to CCK is presumably functionally meaningful.

One of the major actions of PAI-1 is inhibition of the tissue and urokinase plasminogen activators (tPA, uPA) that convert plasminogen to plasmin which in turn digests fibrin, so that PAI-1 is an important player in fibrinolysis. In addition, PAI-1 binds vitronectin, and disrupts interactions with α_v_β_3_ integrins and the uPA receptor (uPAR) leading to biological properties that are independent of tPA or uPA [20]. Previously, uPAR was shown to be expressed by vagal afferent neurons, and knockdown of uPAR expression was associated with decreased capacity of PAI-1 to inhibit CCK effects *in vitro* and *in vivo* [14]. In PAI-1H/Kβ mice there are relatively small changes in plasma PAI-1 compared with C57BL/6 mice, and although not examined directly in the present study, it seems reasonable to suppose that PAI-1 released from gastric epithelial cells in these mice acts locally at uPAR on vagal afferent fibres to decrease sensitivity to CCK in inhibiting gastric emptying.

In obesity, there is evidence for attenuation of the action of CCK in inhibiting gastric emptying [21,22]. Various mechanisms have been discussed including changes in the sensitivity of vagal afferent neurons to CCK with possible roles for the microbiota, interactions with other factors including leptin and ghrelin and neurochemical changes in receptor and neuropeptide gene expression [23–27]. Since there is increased plasma PAI-1 in obesity [18,28] it seems possible that this may also account for depression of responses to CCK. The different proposed mechanisms may not, of course, be mutually exclusive and future work will benefit from systematic dose–response studies. Moreover, in this context it is worth noting that there are multiple populations of vagal afferent neurons that respond to CCK and the precise populations mediating effects on food intake and on gastric emptying remain incompletely characterised [29]. Thus while the neuronal populations targeted by PAI-1 remain to be identified this should be seen in the context of a wider need for the functional characterisation of sub-diaphragmatic vagal afferent neurons.

Multiple gut signals regulate gastric emptying including inhibitory roles for several gut hormones in addition to CCK, e.g. GLP-1 and PYY_3-36_ as well the lipid amide oleylethanolamide, and stimulatory roles for regulatory peptides such as ghrelin [30]. The inhibitory action of PAI-1 against CCK does not preclude interactions with other mediators that could be studied in the future. It is notable that intestinal PAI-1 may be increased after radiation damage [31], in experimental colitis [32], and in intestinal neurons in Crohn's disease [33]. Given that some effects of endogenous CCK on stomach function appear to be mediated by paracrine actions on intestinal vagal afferent fibres while others are mediated by actions of circulating CCK on gastric vagal afferent fibres [29], it would not be surprising if intestinal PAI-1 also played a role in modulating gastric emptying by acting locally on vagal afferent nerve fibres. In some of the circumstances that are associated with increased PAI-1 there are also increases in proinflammatory mediators such as IL1β that enhance vagal responses to CCK [34,35]. The present data therefore suggest a role for gastric PAI-1 in maintaining nutrient delivery to the small intestine in health and disease by counteracting interactions at vagal afferent neurons between CCK and factors that enhance its effects.

## References

[1] Debas H.T., Farooq O., Grossman M.I. (1975). Inhibition of gastric emptying is a physiological action of cholecystokinin. Gastroenterology.

[2] Raybould H.E., Tache Y. (1988). Cholecystokinin inhibits gastric motility and emptying via a capsaicin-sensitive vagal pathway in rats. Am J Physiol.

[3] Forster E.R., Green T., Elliot M., Bremner A., Dockray G.J. (1990). Gastric emptying in rats: role of afferent neurons and cholecystokinin. Am J Physiol.

[4] Fried M., Erlacher U., Schwizer W., Lochner C., Koerfer J., Beglinger C., Jansen J.B., Lamers C.B., Harder F., Bischof-Delaloye A. (1991). Role of cholecystokinin in the regulation of gastric emptying and pancreatic enzyme secretion in humans. Studies with the cholecystokinin-receptor antagonist loxiglumide. Gastroenterology.

[5] Meyer B.M., Werth B.A., Beglinger C., Hildebrand P., Jansen J.B., Zach D., Rovati L.C., Stalder G.A. (1989). Role of cholecystokinin in regulation of gastrointestinal motor functions. Lancet.

[6] Dockray G.J. (2012). Cholecystokinin. Curr Opin Endocrinol Diabetes Obes.

[7] Dockray G.J. (2009). The versatility of the vagus. Physiol Behav.

[8] Barrachina M.D., Martinez V., Wang L., Wei J.Y., Tache Y. (1997). Synergistic interaction between leptin and cholecystokinin to reduce short-term food intake in lean mice. Proc Natl Acad Sci U S A.

[9] Date Y., Toshinai K., Koda S., Miyazato M., Shimbara T., Tsuruta T., Niijima A., Kangawa K., Nakazato M. (2005). Peripheral interaction of ghrelin with cholecystokinin on feeding regulation. Endocrinology.

[10] de Lartigue G., Lur G., Dimaline R., Varro A., Raybould H., Dockray G.J. (2010). EGR1 is a target for cooperative interactions between cholecystokinin and leptin, and inhibition by ghrelin, in vagal afferent neurons. Endocrinology.

[11] Norsett K.G., Steele I., Duval C., Sammut S.J., Murugesan S.V., Kenny S., Rainbow L., Dimaline R., Dockray G.J., Pritchard D.M., Varro A. (2011). Gastrin stimulates expression of plasminogen activator inhibitor-1 in gastric epithelial cells. Am J Physiol Gastrointest Liver Physiol.

[12] Kenny S., Duval C., Sammut S.J., Steele I., Pritchard D.M., Atherton J.C., Argent R.H., Dimaline R., Dockray G.J., Varro A. (2008). Increased expression of the urokinase plasminogen activator system by *Helicobacter pylori* in gastric epithelial cells. Am J Physiol Gastrointest Liver Physiol.

[13] Keates A.C., Tummala S., Peek R.M., Csizmadia E., Kunzli B., Becker K., Correa P., Romero-Gallo J., Piazuelo M.B., Sheth S., Kelly C.P., Robson S.C., Keates S. (2008). *Helicobacter pylori* infection stimulates plasminogen activator inhibitor 1 production by gastric epithelial cells. Infect Immun.

[14] Kenny S.H., Gamble J., Lyons S., Vlatkovic N., Dimaline R., Varro A., Dockray G.J. (2013). Gastric expression of plasminogen activator inhibitor (PAI)-1 is associated with hyperphagia and obesity in mice. Endocrinology.

[15] Berkenpas M.B., Lawrence D.A., Ginsburg D. (1995). Molecular evolution of plasminogen activator inhibitor-1 functional stability. EMBO J.

[16] Forster E.R., Dockray G.J. (1992). The role of cholecystokinin in inhibition of gastric emptying by peptone in the rat. Exp Physiol.

[17] Green T., Dimaline R., Peikin S., Dockray G.J. (1988). Action of the cholecystokinin antagonist L364,718 on gastric emptying in the rat. Am J Physiol.

[18] Landin K., Stigendal L., Eriksson E., Krotkiewski M., Risberg B., Tengborn L., Smith U. (1990). Abdominal obesity is associated with an impaired fibrinolytic activity and elevated plasminogen activator inhibitor-1. Metabolism.

[19] Boehm J.R., Kutz S.M., Sage E.H., Staiano-Coico L., Higgins P.J. (1999). Growth state-dependent regulation of plasminogen activator inhibitor type-1 gene expression during epithelial cell stimulation by serum and transforming growth factor-beta1. J Cell Physiol.

[20] Dellas C., Loskutoff D.J. (2005). Historical analysis of PAI-1 from its discovery to its potential role in cell motility and disease. Thromb Haemost.

[21] Little T.J., Horowitz M., Feinle-Bisset C. (2007). Modulation by high-fat diets of gastrointestinal function and hormones associated with the regulation of energy intake: implications for the pathophysiology of obesity. Am J Clin Nutr.

[22] Covasa M. (2010). Deficits in gastrointestinal responses controlling food intake and body weight. Am J Physiol Regul Integr Comp Physiol.

[23] Kentish S., Li H., Philp L.K., O'Donnell T.A., Isaacs N.J., Young R.L., Wittert G.A., Blackshaw L.A., Page A.J. (2012). Diet-induced adaptation of vagal afferent function. J Physiol.

[24] de Lartigue G., Barbier dlS, Espero E., Lee J., Raybould H.E. (2011). Diet-induced obesity leads to the development of leptin resistance in vagal afferent neurons. Am J Physiol Endocrinol Metab.

[25] Raybould H.E. (2012). Gut microbiota, epithelial function and derangements in obesity. J Physiol.

[26] Dockray G.J., Burdyga G. (2011). Plasticity in vagal afferent neurones during feeding and fasting: mechanisms and significance. Acta Physiol (Oxf).

[27] Daly D.M., Park S.J., Valinsky W.C., Beyak M.J. (2011). Impaired intestinal afferent nerve satiety signalling and vagal afferent excitability in diet induced obesity in the mouse. J Physiol.

[28] Alessi M.C., Poggi M., Juhan-Vague I. (2007). Plasminogen activator inhibitor-1, adipose tissue and insulin resistance. Curr Opin Lipidol.

[29] Okano-Matsumoto S., McRoberts J.A., Tache Y., Adelson D.W. (2011). Electrophysiological evidence for distinct vagal pathways mediating CCK-evoked motor effects in the proximal versus distal stomach. J Physiol.

[30] Dockray G.J. (2009). Cholecystokinin and gut-brain signalling. Regul Pept.

[31] Abderrahmani R., Francois A., Buard V., Tarlet G., Blirando K., Hneino M., Vaurijoux A., Benderitter M., Sabourin J.C., Milliat F. (2012). PAI-1-dependent endothelial cell death determines severity of radiation-induced intestinal injury. PLoS One.

[32] Hyland N.P., Chambers A.P., Keenan C.M., Pittman Q.J., Sharkey K.A. (2009). Differential adipokine response in genetically predisposed lean and obese rats during inflammation: a role in modulating experimental colitis?. Am J Physiol Gastrointest Liver Physiol.

[33] Laerum O.D., Illemann M., Skarstein A., Helgeland L., Ovrebo K., Dano K., Nielsen B.S. (2008). Crohn's disease but not chronic ulcerative colitis induces the expression of PAI-1 in enteric neurons. Am J Gastroenterol.

[34] Kurosawa M., Uvnas-Moberg K., Miyasaka K., Lundeberg T. (1997). Interleukin-1 increases activity of the gastric vagal afferent nerve partly via stimulation of type A CCK receptor in anesthetized rats. J Auton Nerv Syst.

[35] Bucinskaite V., Kurosawa M., Miyasaka K., Funakoshi A., Lundeberg T. (1997). Interleukin-1beta sensitizes the response of the gastric vagal afferent to cholecystokinin in rat. Neurosci Lett.

